# The Influence of Probiotic Supplementation on the Obesity Indexes, Neuroinflammatory and Oxidative Stress Markers, Gut Microbial Diversity, and Working Memory in Obese Thai Children

**DOI:** 10.3390/foods12213890

**Published:** 2023-10-24

**Authors:** Suchanat Khongtan, Bhagavathi Sundaram Sivamaruthi, Subramanian Thangaleela, Periyanaina Kesika, Muruganantham Bharathi, Sasithorn Sirilun, Thiwanya Choeisoongnern, Sartjin Peerajan, Phakkharawat Sittiprapaporn, Chaiyavat Chaiyasut

**Affiliations:** 1 Innovation Center for Holistic Health, Nutraceuticals, and Cosmeceuticals, Faculty of Pharmacy, Chiang Mai University, Chiang Mai 50200, Thailandsivamaruthi.b@cmu.ac.th (B.S.S.); kesika.p@cmu.ac.th (P.K.);; 2 Department of Pharmaceutical Sciences, Faculty of Pharmacy, Chiang Mai University, Chiang Mai 50200, Thailand; 3 Office of Research Administration, Chiang Mai University, Chiang Mai 50200, Thailand; 4 Neuropsychological Research Laboratory, Neuroscience Research Center, School of Anti-Aging and Regenerative Medicine, Mae Fah Luang University, Bangkok 10110, Thailand; 5 Health Innovation Institute, Chiang Mai 50200, Thailand

**Keywords:** obesity, *Lactobacillus paracasei* HII01, gut microbiome, neuroinflammation, working memory, brain wave

## Abstract

Obesity is a worldwide health problem with a complex interaction between gut microbiota and cognition. Several studies have demonstrated that probiotic treatments improve characteristics linked to obesity. The present study aimed to evaluate the effects of probiotic supplementation on the obesity indexes, inflammatory and oxidative stress markers, gut microbiota, and working memory in obese children. Ten obese children were assigned to receive the probiotics (8 × 10^9^ CFU of *Lactobacillus paracasei* HII01 and *Bifidobacterium animalis* subsp. *lactis*) for 12 weeks. Demographic data were recorded. Urine and fecal samples were collected to evaluate biomarkers related to obesity and cognition. Behavioral working memory was assessed using the visual n-back test. Electroencephalography was employed to measure electrical activity during the visual n-back test. All parameters were evaluated at the baseline and after 12 weeks. The results revealed that probiotic supplementation significantly altered some gut microbial metabolites, gut microbiota, total antioxidant capacity, and neuroinflammatory markers. However, no significant changes were observed in the visual n-back test or electroencephalographic recordings after 12 weeks. In conclusion, the use of probiotics might be an alternative treatment that could improve the gut microbial ecosystem and microbial metabolites, as well as host antioxidant and neuroinflammation levels. The preliminary results indicated that further detailed prolonged studies are needed in order to determine the beneficial effects of the studied probiotics.

## 1. Introduction

Obesity is a global health threat. It is multifactorial chronic disease characterized by excessive fat deposition in the body, and is related to the development of other complications, such as insulin resistance, increased risk of diabetes, dyslipidemia, hypertension, cardiovascular diseases, and other related disorders [1]. The World Health Organization (WHO) set body mass index as a simplified marker for measuring and classifying the obese condition [2]. Obesity affects both children and adults and has reached epidemic levels in developing countries. Childhood obesity is associated with an early onset of non-communicable diseases, such as cardiovascular diseases and diabetes. Lifestyle, food practices involving a high intake of excessive sugar-containing foods, increased caloric intake with reduced physical activity, racial and ethnic group-related gene–nutrition interactions, and environmental factors are some of the major factors that cause obesity [3]. Obesity in childhood increasingly affects a child’s physical, mental, and emotional health and presents a high risk for comorbid conditions, including metabolic, pulmonary, hepatic, renal, orthopedic, neurological, and cardiovascular conditions [4].

In addition to various other etiological reasons for obesity, one of the major factors is the gut microbiota. Alterations in the gut microbial composition and functions result in obesity and obesity-related metabolic disorders [5]. The gut microbiota exerts many important functions, including energy homeostasis, immune functions, the ingestion of undigested carbohydrates, the retention of electrolytes and minerals, the balancing of gut motility, and the production of micronutrients [6,7]. The gut microbiota also regulates and instigates inflammation during metabolic ailments [7]. The changes in the ratios of Firmicutes, Bacteroidetes, and certain short-chain fatty acid (SCFA)-producing bacterial genera contribute to obesity [5]. Additionally, studies have described the links between bacterial composition and the obese condition. The transplantation of gut microbiota from wild mice into germ-free mice without increasing food intake resulted in increased adiposity [8], and the transplanted mice ingested more energy from their diets and excreted less [9].

A comparative study of lean and obese mice stated that obese mice show a 50% reduction in *Bacteroidetes*, increased abundance of Firmicutes, and increased concentration of cecal microbial fermentation products like acetate and butyrate [9,10]. The gut microbiomes of obese mice revealed the presence of genes involved in energy harvesting and metabolism [11]. Another study in obese humans revealed a reduced Bacteroidetes/Firmicutes ratio and enhanced *Proteobacteria* abundance [12,13]. Obesity is associated with increased *Lactobacillus* and *Staphylococcus*, whereas normal weight is associated with increased abundances of *Bifidobacterium*, *Methanobrevibacter*, and *Bacteroidetes* [14,15]. Thus, the alterations in the gut microbiome are profoundly related to the incidence of obesity, as they influence host metabolic functions through intricate signaling mechanisms, inflammatory and metabolic pathways, and energy deposition in fat stores [16].

Inflammation is a common biological response of the immune system against pathogens, damaged cells, and endotoxins. Low-grade chronic inflammation is observed in obesity [17]. The low-grade chronic systemic inflammation in adipose tissue is critical in obesity-related complications. The adipose tissue in visceral areas actively releases adipocytokines and adipokines. The activation of the innate immune system in adipose tissue initiates the pro-inflammatory status and oxidative stress, which induce the systemic response, resulting in obesity and other chronic metabolic diseases like diabetes mellitus, cardiovascular diseases, and other metabolic syndromes [18].

Adipose tissue dysfunction produces systemic oxidative stress. The biomarkers of oxidative stress are directly proportional to BMI, body fat, and triglyceride levels [19]. Adipose tissue is a center for inflammatory factors like TNF-α and IL-6 and oxidative markers like nicotinamide adenine dinucleotide phosphate and reactive oxygen species [20]. Oxidative stress is an important sign of the pathophysiology of metabolic diseases, including obesity. The total antioxidant capacity (TAC) in plasma and tissues changes during oxidative stress, and the measure of TAC could be a suitable marker for studying metabolic dysfunctions. A consistent relationship between TAC and obesity indexes was found [20]. Researchers observed that TAC is independently related to BMI and waist-to-hip ratio. The obese or overweight individuals had lower TAC compared to normal-weight individuals. The study concluded that obesity and central adiposity conditions may reduce TAC, irrespective of age and lifestyle preferences [20]. Obesity due to chronic overnutrition causes inflammation in the central nervous system. The increased metabolic functions and metabolites that flux into the brain due to obesity and overnutrition can induce a stress response including blood–brain barrier disruption, and recruit inflammatory immune cells, leading to neuroinflammation [21].

Long-term overweight conditions starting in childhood are associated with reduced urinary gut bacterial indole-3-acetic acid and many other urinary amino acids. Indole metabolites and their producers, gut bacteria, play a prominent role in obesity-related inflammatory reactions [22]. Obese individuals with a history of being overweight consistently from childhood had lower levels of urinary indole-3-acetic acid than others. The 5-hydroxyindole-3-acetic acid was positively associated with C-reactive proteins in late adolescence, which is the marker of inflammation [22]. Obesity increases the risk of developing neurogenerative disease with cognitive decline. Quinolinic acid (QA), a neuroinflammatory neurotoxin secreted by microglia during inflammation, is one of the causes of the pathogenicity of neurogenerative processes. The involvement of QA in the mechanism of cognitive decline in obesity has also been studied [23].

Working memory (WM) is one of the essential executive functions in a set of cognitive processes [24]. WM is a necessary cognitive ability that greatly affects one’s daily life. Problems with WM are associated with poor quality of life due to neurodevelopmental disorders and other illnesses, particularly major risk factors that negatively impact a child’s capacity for learning and academic performance [25,26].

Studies found that obese people frequently suffer from poor cognitive function in a part of WM, which involves chronic, low-grade inflammation that results from changes in the composition of the gut microbiota and adipose tissue [27,28,29].

Cognitive ability throughout childhood and adolescence is influenced by many factors, including diet [30]. There is mounting evidence that normal cognitive function is associated with a healthy gut microbiota. In a recent double-blind, randomized, placebo-controlled trial by Papalini et al. [31], multi-strain probiotics improved WM performance in healthy female participants with acute stress compared to the placebo group. The result was linked to the neural mechanisms involved in the frontal cortex during cognitive control [31]. Similarly, Handajani et al. [32] investigated the effects of probiotic supplementation on cognitive impairment in older adults. The cognitive functions of language, visuospatial function, and memory significantly improved in the probiotic group, indicating that probiotics could be an effective alternative for improving various cognitive and psychological conditions [32].

Accordingly, the present study aimed to evaluate the effects of the probiotic supplements (*Lactobacillus paracasei* HII01 and *Bifidobacterium animalis* subsp. *lactis*) on the obesity indexes, inflammatory markers, gut microbiota, and working memory in obese Thai children.

## 2. Materials and Methods

The study protocols were approved by the Ethical Committee of the Mae Fah Luang University, Chiang Rai, Thailand (approval number: EC 21059-20 dated 7 October 2021), and the study followed the Good Clinical Practices and the guidelines established in the Declaration of Helsinki. The purpose and methodology of the study were explained to the participants’ parents. Parental consent and participant assent were received before the commencement of the study. 

### 2.1. Study Group 

**Figure 1:**
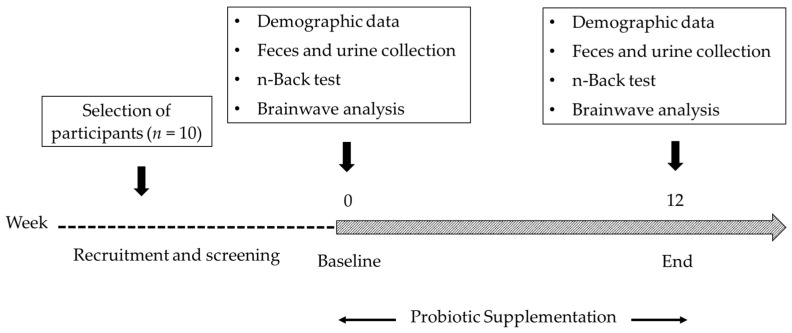
The timeline of the study.

Obese children aged 7 to 12 years, with a BMI ≥ 25 kg/m^2^, from the local village (Muang District, Chiang Mai) were included in the study. Participants with neurological conditions or those who had taken antibiotic or probiotic products in the previous 14 days were excluded from the study. Among the 23 enrolled subjects, 10 participants (*n* = 10; 6 males and 4 females) were included in the study after screening. Power analysis was performed using the STATA program to validate the number of study subjects. The analysis showed that *n* = 7 was sufficient, with a power value of 0.8000 and an estimated dropout of 30–50%. Thus, we used 10 subjects in this study. The participants were assigned to receive probiotics for 12 weeks. Urinary and fecal samples were collected from the participants at the baseline (week 0) and after treatment (week 12) of supplementation. Participants were asked to follow the assigned follow-up visits without absence. The changes in their parameters and microbial compositions were studied. The study protocol is described in Figure 1. 

### 2.2. Probiotic Supplementation

Aluminum foil sachets containing 8 × 10^9^ CFU of *L. paracasei* HII01 (2 × 10^9^ CFU) and *B. animalis* subsp. *lactis* (6 × 10^9^ CFU) were given to the participants and they were asked to consume the probiotics once daily, before lunch with a glass of water or fruit juice, for 12 weeks. The probiotics were prepared and provided by Lactomason Co., Ltd. (Jinju, Gyeongsangnam-do, Republic of Korea). Participants were instructed to store the sachets away from strong sunlight in a dry environment, between 2 and 8 °C. Participants were asked not to change their usual physical activity levels, nutrition, or lifestyle habits. During the study, they were informed to avoid consuming other probiotics, fermented foods, or other dietary supplements.

### 2.3. Demographic Assessments

Demographic and clinical data were collected from the participants. A weighing scale (Picooc^®^, Model S1 Pro, Beijing, China) was employed to determine the individual’s body weight, body mass index (BMI), body fat, and visceral fat. The arm, waist, and hip circumferences were assessed with a tape measure, and the waist/hip ratio was computed (Table 1).

### 2.4. Measurement of Gut Microbial Metabolites

Fecal samples were used to identify SCFAs using high-performance liquid chromatography (HPLC). The analysis was conducted as detailed previously. A Shimadzu-HPLC system (Shimadzu, Kyoto, Japan) with a UV-Vis detector and a Shodex SUGAR SH1011 column (Showa Denko K.K., Tokyo, Japan) was used. Fecal samples were also examined for putrefaction biomarkers (indole, cresol, and skatole) using the HPLC with a C18 column (Showa Denko K.K., Tokyo, Japan). SCFAs and putrefaction were quantified by comparing them with the standard curve, and the results were expressed as µmol/g sample [33].

### 2.5. Measurement of Oxidative Stress Markers

#### 2.5.1. Total Antioxidant Capacity (TAC)

A DPPH (2,2-diphenyl-1-picryl-hydrazyl) assay was performed to assess the total antioxidant capacity (TAC) in urine samples with the modified method [34,35]. Briefly, 5 µL of urine sample was added to 95 µL of 10 mM PBS (phosphate buffered saline, pH 7.4) and 100 µL of 0.1 mM DPPH in methanol in a 96-well plate. The reactions were kept in the dark for 20 min at room temperature. Absorbance was measured using a microplate reader (SpectraMax M3, Molecular Devices LLC, San Jose, CA, USA) at 520 nm. A standard curve was generated using the percentage of inhibition against vitamin C at a concentration of 0.125 to 8 mM. The results were expressed as the vitamin C equivalent antioxidant capacity (VCEAC) in millimolars (mM).

#### 2.5.2. Lipid Peroxidation

A thiobarbituric acid reactive substances (TBARS) assay was performed to determine malondialdehyde (MDA) levels [36]. Briefly, 50 µL of urine sample was mixed with 50 µL of 0.05% BHT (Butylated hydroxytoluene), 150 µL of 0.1125 N HNO3 (Nitric acid), and 150 µL of 42 mM TBA (Thiobarbituric acid). This mixture was incubated at 95 °C for 1 h and then cooled on ice for 5–10 min. Then, the solution was centrifuged at 1500 rpm at room temperature for 5 min, and 200 μL of the sample was transferred to a 96-well plate. Absorbance was measured using a microplate reader (SpectraMax M3, Molecular Devices LLC, San Jose, CA, USA) at 532 nm. A standard curve was generated using absorbance against different MDA standard concentrations (0.15 to 20 µM). The results were presented in micromolars (µM).

### 2.6. Measurement of Neuroinflammatory Markers 

The participants’ urine samples were collected in a clean container before their first meal. Urinary quinolinic acid (QA) was determined using the enzyme-linked immunosorbent assay (ELISA) according to the manufacturer’s recommendations (Five photon Biochemicals™, San Diego, CA, USA). Urinary 5-hydroxyindoleacetic acid (5-HIAA) was measured using the competitive ELISA following the manufacturer’s recommendations (Immusmol, Bordeaux, France). The ratio of QA/5-HIAA was also computed [37].

### 2.7. Next-Generation Sequencing (NGS)

Fecal genomic DNA was isolated using the QIAamp PowerFecal DNA Kit (Catalog no. 12830-50, QIAGEN, Hilden, Germany), following the manufacturer’s recommendations. The DNA sequencing was conducted by the Omics Sciences and Bioinformatics Center, Faculty of Science, Chulalongkorn University, Thailand, as detailed in our previous study [38].

### 2.8. Assessment of Working Memory

Working memory was measured with ERP analysis via the oddball visual paradigm and the neuropsychological test via a computer interface. Response times were based on manual keypad or mouse entry, and brain ERP detection was performed simultaneously [39].

#### 2.8.1. Neuropsychological Testing

**Figure 2:**
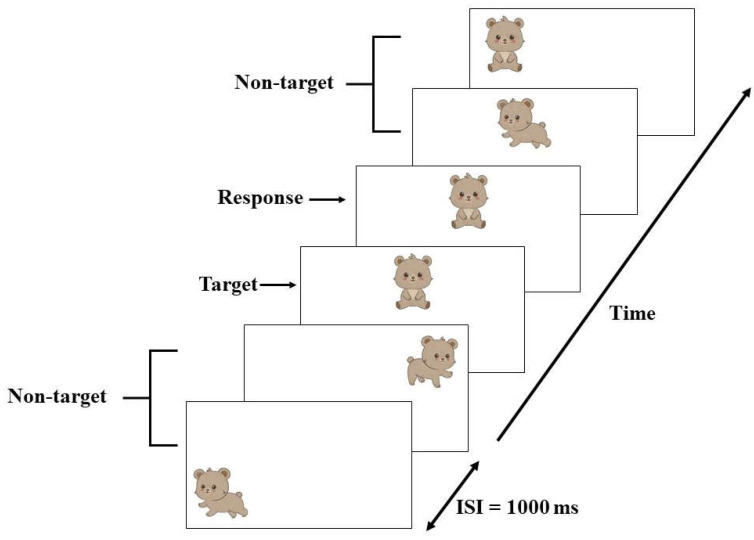
The visual n-back task [41].

The neuropsychological test used in this study was the visual n-back test. In this visual n-back test, three sets of bear pictures were presented to the participant. According to this test, a list of moving bear positions was presented to the participant one at a time, for example, at 75°, 90°, and 180°. The participant was requested to memorize only the bear at the 90° position as it moved around. The participants were required to recall the bear at the 90° position when it returned to the same place. During this task, the bear at the 90° position that served as the n-back signal was presented on a monitor at around 150 cm from the participant’s eyes. The bear at the 90° position represented the target n-back condition with a 25 percent probability, and the other positions represented the non-target condition with a 75 percent probability of appearance (Figure 2).

Participants completed the tests using the e-Prime version 3.0 software (Psychology Software Tools, Inc., Pittsburgh, USA), displayed around 150 cm from their eyes on a computer screen. The visual version of the bear series was shown to all participants using a computer linked to a button keypad. The pictures of targets (the bear seated in the upper-center position: 90° position) and non-targets (the bear in any position, running or walking: 75° and 180° positions) flashed on the screen sequentially, one at a time, for 1500 milliseconds (ms) each. Targets had an appearance probability of 25%, whereas non-targets had a probability of 75%. When the target appeared, the participants were told to press the button as quickly and accurately as they could using the finger of the dominant hand. The participants were also told to disregard the button whenever a non-target appeared. In the visual n-back test, the target bear was repeatedly displayed twice, and the participants were instructed to remember it and respond immediately if it reappeared. The participants completed the test in approximately 5–10 min. For analysis, the key press responses of the participants were used to determine their reaction latencies and response accuracy or error. Correct answers were provided by participants who depressed the button that matched the target stimuli while rejecting the non-target ones. On the other hand, it was considered an incorrect answer if they pressed a button on stimuli that did not match the target ones. Furthermore, participants who did not respond when the target stimuli appeared were considered to have made an omission error. The percentages of accuracy and error were counted, and the reaction times in milliseconds (ms) were averaged across all participants [40].

#### 2.8.2. Event-Related Potential (ERP) Recording Procedure

EEG/ERP recordings were based on signals detected through the scalp with a wearable, multi-electrode array cap (Electro-cap, eego^TM^, ANT Neuro, PE Hengelo, The Netherlands). A 32-channel set of electrodes (Fp1, Fpz, Fp2, F7, F3, Fz, F4, F8, FC5, FC1, FC2, FC6, T7, C3, Cz, C4, T8, CP5, CPz, CP1, CP2, CP6, P7, P3, Pz, P4, P8, O1, O2, Oz, M1, and M2) was pre-mounted within the elastic Electro-Cap (Waveguard™ original EEG cap, ANT Neuro, PE Hengelo, The Netherlands) according to the International 10–20 Electrode Positioning System. The Waveguard™ EEG cap (ANT Neuro, PE Hengelo, The Netherlands) is easy to implement, using very thin electrode wires, and the flexible, breathing cap fabric enables comfortable recordings even over a longer period. Between the electrodes Fz and Cz, a ground electrode was attached. The M1 and M2 reference electrodes were placed on ipsilateral mastoids, with the Fp1 and Fp2 electrodes employed for ocular artifact detection. The resistance of the electrodes was less than 10 kΩ. With a 0.05 to 100 Hz bandpass, the EEG signals were amplified and captured at 500 Hz, and live signal data were saved to a hard disk for offline processing. A 0.1–30 Hz band pass was then used to filter recorded ERPs digitally. The average epoch calculated was 500 ms, and the baseline was 100 ms before the commencement of the presenting stimuli. All neural and ocular artifacts were removed from the continuous EEG before the extraction of ERP waves. The epochs were extracted from the EEG-free artifact starting from 100 ms pre-stimulus and continuing to 500 ms post-stimulus. Baseline correction was also applied to each epoch, with any changes in voltage below 0.1 μV or above 70 μV rejected from further analysis [39].

After registration, the data were re-referenced offline to the common average montage, followed by the correction and rejection of artifacts. EEG epochs with absolute amplitudes greater than 100 µV were automatically flagged and removed from further investigation. Before averaging, all channels were subjected to artifact rejection with a threshold of ±100 µV. ERP waveforms were generated to investigate the ERP components where the non-target stimuli evoked reactions of frequent stimuli, presenting a 75% probability. Infrequent stimuli for target conditions were presented randomly with a probability of 25% (oddball paradigm). The interstimulus interval (ISI) was 1000 ms. The amplitude (μV) and latency (ms) of the ERP signals were measured. The total recording time was 5 min for each visual n-back test. The positive peak between 250 and 400 ms was defined as P300. Both latencies and amplitudes of the targets were recorded and analyzed. All ERP analyses were performed using standardized Low-resolution Brain Electromagnetic Tomography (sLORETA) analysis software featuring source reconstruction, signal analysis, and MRI processing tools [42].

The global field power (GFP) peak metric was used to assess a brain electric field map’s electric strength (hilliness), independent of its spatial layout. The spatial standard deviation of all voltage estimations was based on one spontaneous EEG map. A steep potential map would have a higher GFP peak than a flat one. The GFP described above was self-contained [43,44]. The spatial standard deviation of GFP quantifies the activity at each time point in the field, resulting in a reference-independent descriptor of the potential field. The occurrence times of GFP maxima were used to determine the latencies of visually evoked potential components, which became complimentary over time [45]. This was performed by averaging the ERPs from all scalp channels while excluding electrooculographic channels. Participants’ mean and grand mean GFP peak amplitudes were computed [43,44,45,46] and statistically analyzed per the cognitive function tests.

### 2.9. Statistical Analysis

Descriptive data were presented as the mean ± standard error of the mean (SEM) for continuous outcomes, or as an absolute number and percentage for categorical outcomes. The outcomes of an individual were compared before and after the intervention. A paired *t*-test analyzed the normally distributed data. The Wilcoxon signed-rank test was used to analyze the skewed data. A *p*-value of less than 0.05 was considered statistically significant (two-tailed). The statistical analysis for this study was completed with STATA version 15.1 for Windows (StataCorp, College Station, TX, USA).

## 3. Results

### 3.1. The Effect on Obesity Index, Gut Microbial Metabolites, Oxidative Stress, and Neuroinflammatory Markers

The subjects did not report any adverse effects during the study period. Parameters such as body weight, BMI, body, and visceral fats were measured at baseline and after 12 weeks of the study. No statistically significant differences were observed in the studied obesity indexes between the samples collected from the baseline and after treatment (Table 2). SCFAs, including butyric and propionic acids, were significantly elevated after 12 weeks of probiotic supplementation. We observed the changes in the levels of lactic and acetic acids, cresol, indole, and skatole after 12 weeks of probiotic supplementation, but they were also statistically insignificant (Table 2). The level of TAC was significantly increased after probiotic supplementation compared to the baseline (Table 2). Neuroinflammatory markers, such as QA and 5-HIAA, showed significant changes from the baseline to the treatment phase. QA and 5-HIAA were initially significantly reduced, and they enhanced after treatment with probiotic supplementation. The QA/5-HIAA ratio was significantly reduced after probiotic treatment compared to the baseline (Table 2).

### 3.2. Bacterial Sequencing

The average numbers of sequence’ reads and non-chimeric sequences of baseline (week 0) samples were 47,662 and 38,750, respectively. The average numbers of sequence reads and non-chimeric sequences of treatment samples (week 12) were 50,059 and 38,985, respectively (Table 3).

#### 3.2.1. Alpha Diversity

**Figure 3:**
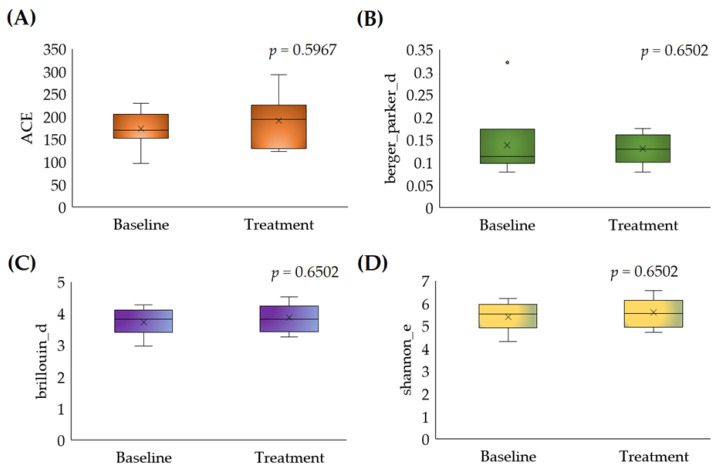
Alpha diversity estimation between the baseline and post-treatment with the parameters (**A**) ACE, (**B**) Berger–Parker, (**C**) Brillouin, and (**D**) Shannon entropy. Note: ACE: abundance-based coverage estimator. The circle dot indicates the outlier sample.

The alpha diversity was measured for details regarding the significant differences in species abundances and their associated diversity. The abundance coverage estimator (ACE) metric was implemented to calculate and visualize the community richness differences between the baseline and treatment samples. The results stated that the community richness was not significantly different (*p* = 0.5967) (Figure 3A). Also, a Berger–Parker analysis was performed to identify the significant differences in the relative richness of the abundant species. The results showed no significant changes between the baseline and treatment samples regarding the mean of species relative richness (*p* = 0.6502) (Figure 3B).

Similarly, the Brillouin parameter was analyzed for the significant differences in species diversity, and there were no significant differences observed in species diversity between the baseline and treatment samples (*p*-value = 0.6502) (Figure 3C). Further, Shannon entropy was calculated to identify the significant differences in the taxonomical abundances and evenness between the baseline and treatment samples. The results showed that the taxonomical abundances and evenness were similar in the baseline and treatment samples (*p*-value = 0.6502) (Figure 3D).

#### 3.2.2. Beta Diversity

**Figure 4:**
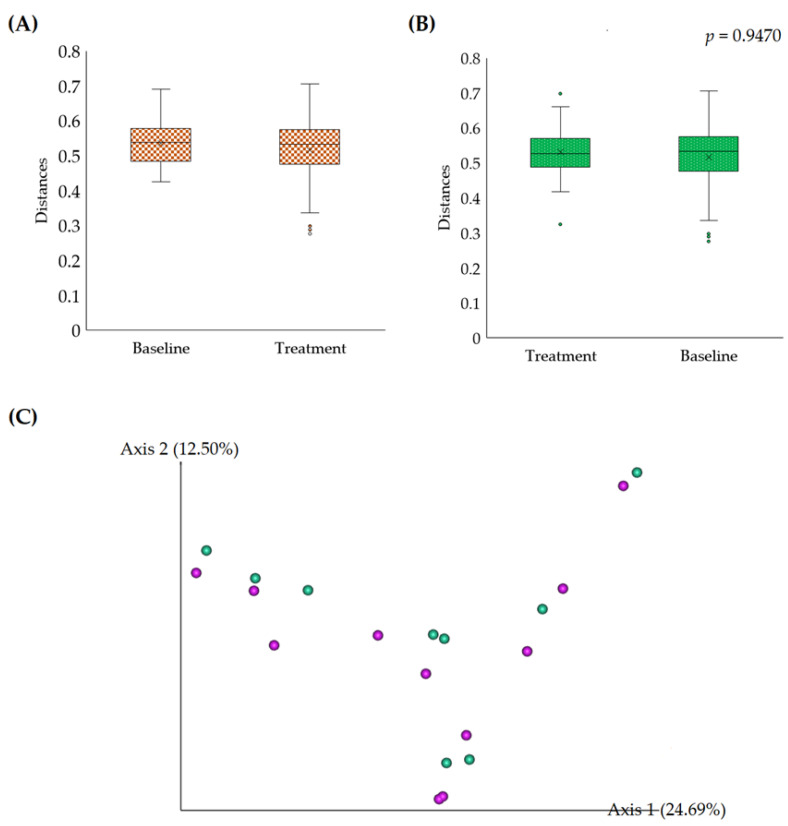
Beta diversity was analyzed using the PERMANOVA (**A**) distances to baseline, (**B**) distances to treatment, and (**C**) PCoA 2-dimensional plot based on the Unirac unweighted analysis of group significance. The circle dot indicates the outlier samples. The pink and green circles indicate the baseline and treatment samples.

Beta diversity was estimated using the permutational multivariate analysis of variance (PERMANOVA) test and principal coordinate analysis (PCoA) using the Unirac unweighted analysis of group significance. The PERMANOVA was performed with 999 permutations, and the observed *p*-value was 0.9767. The outcomes indicated no significant distance differences between the samples (Figure 4A,B). Likely, the baseline and treatment samples were not significantly separated in the PCoA plot, which indicates the resemblance of the microbial load in the baseline and treatment samples (Figure 4C).

#### 3.2.3. Taxonomical Assignment and Quantification 

**Figure 5:**
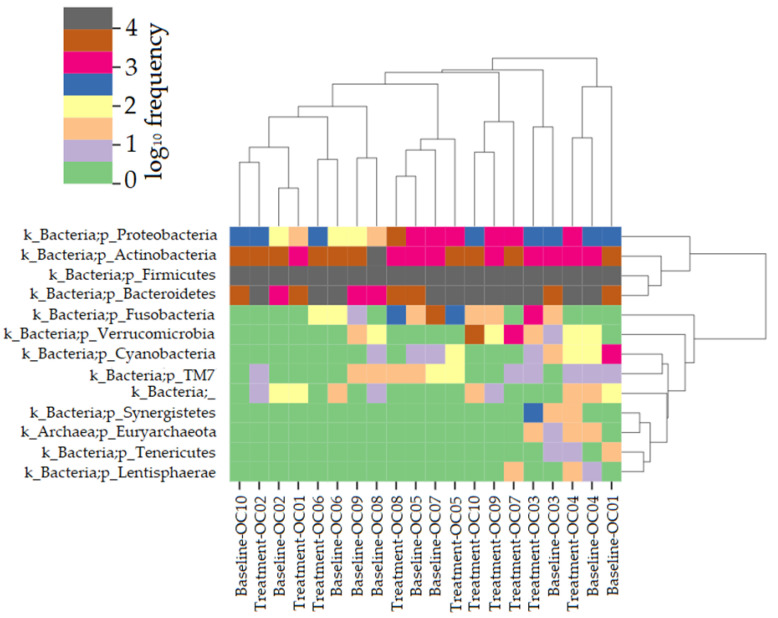
The taxonomy was estimated for the samples from the baseline and after 12 weeks of treatment. The predicted total phylum diversity was represented as a heatmap with log_10_ frequency (0–4).

The bacterial taxonomy was predicted for the baseline and treatment samples and visualized as a heat map. The phyla Proteobacteria, Actinobacteria, Firmicutes, Bacteriodetes, Fusobcateria, Verrucomicrobia, Cyanobacteria, TM7, Synergistetes, Euryarchaeota, Tenericutes, and Lentisphaerae were detected (Figure 5).

#### 3.2.4. Phylum Quantification

**Figure 6:**
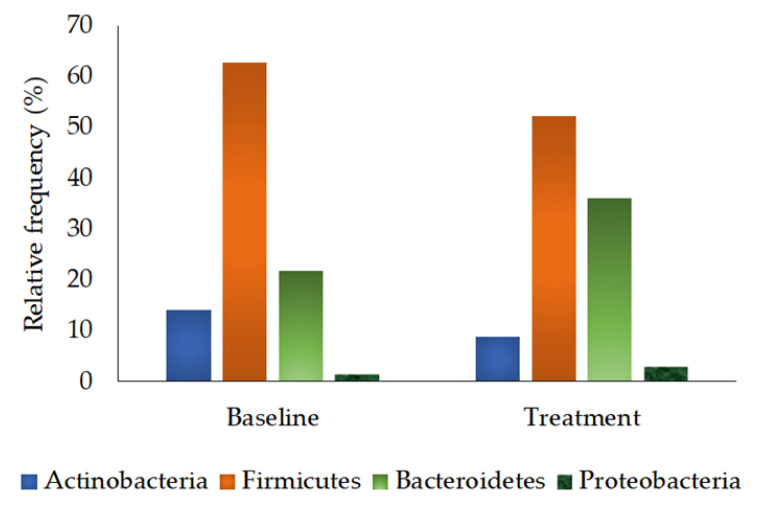
The predominant phyla in the baseline and treatment samples (≥70% in baseline or treatment) were calculated and compared. The significant difference was estimated for the phyla detected between the baseline and treatment samples based on the Wilcoxon signed-rank test, with a statistical significance of *p* ≤ 0.05. No statistically significant differences were observed between the samples.

The phyla Actinobacteria, Firmicutes, Bacteroidetes, and Proteobacteria, were found dominantly (≥70%) in the baseline and treatment samples based on the calculated relative frequency (Figure 6). In detail, the phyla Firmicutes (Baseline RF = 62.69 ± 5.09%; Treatment RF = 52.27 ± 5.33%), Actinobacteria (Baseline RF = 14.12 ± 5.25%; Treatment RF = 8.74 ± 1.80%), Bacteroidetes (Baseline RF = 21.72 ± 5.68%; Treatment RF = 36.00 ± 4.93%), and Proteobacteria (Baseline RF = 1.47 ± 0.71%; Treatment RF = 2.99 ± 1.15%) were detected. Phylum Firmicutes was detected with higher frequency than the other three phyla in the baseline and treatment samples. A Wilcoxon signed-rank test was used to determine the significance of differences, and the results showed no significant changes between the baseline and treatment samples (Table 4).

#### 3.2.5. Genera Quantification

**Figure 7:**
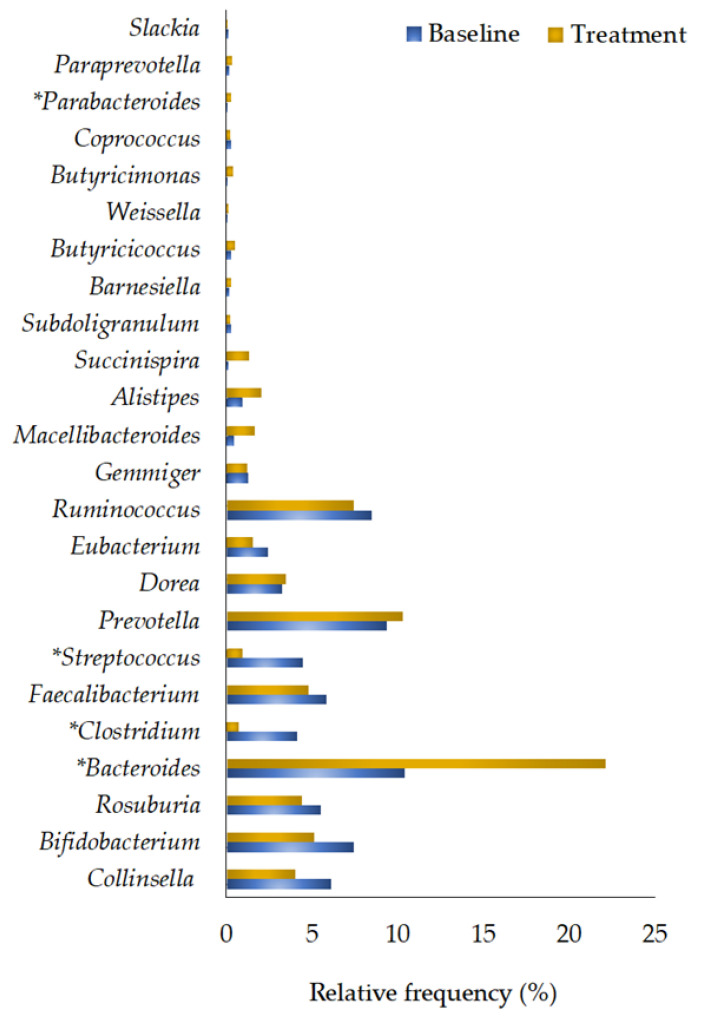
The predominant genera in the baseline and treatment samples (≥70% in baseline or treatment) were calculated and compared. A significant difference was estimated for the detected genera between the baseline and treatment samples based on the Wilcoxon signed-rank test, with a statistical significance of *p* ≤ 0.05. Note: *: significantly differed (*p* ≤ 0.05) compared to the baseline samples.

The genera *Collinsella*, *Bifidobacterium*, *Roseburia*, *Bacteroides*, *Faecalibacterium*, *Streptococcus*, *Prevotella*, *Dorea*, *Eubacterium*, *Ruminococcus*, *Gemmiger*, *Clostridium*, *Macellibacteroides*, *Alistipes*, *Succinispira*, *Subdoligranulum*, *Barnesiella*, *Butyricicoccus*, *Weissella*, *Butyricimonas*, *Coprococcus*, *Parabacteroides*, *Paraprevotella*, and *Slackia* were observed in both the baseline and treatment samples (Figure 7). Significant differences were found in the abundances of *Bacteroides* (Baseline RF = 10.39 ± 3.93%; Treatment RF = 22.11 ± 4.23%; *p* = 0.009), *Parabacteroides* (Baseline RF = 0.04 ± 0.02%; Treatment RF = 0.29 ± 0.14%; *p* = 0.018), *Clostridium* (Baseline RF = 3.23 ± 1.08%; Treatment RF = 0.67 ± 0.12%; *p* = 0.018), and *Streptococcus* (Baseline RF = 4.48 ± 1.26%; Treatment RF = 0.98 ± 0.25%; *p* = 0.017) in the treated samples compared to the baseline values (Figure 7; Table 4).

#### 3.2.6. Species Quantification

**Figure 8:**
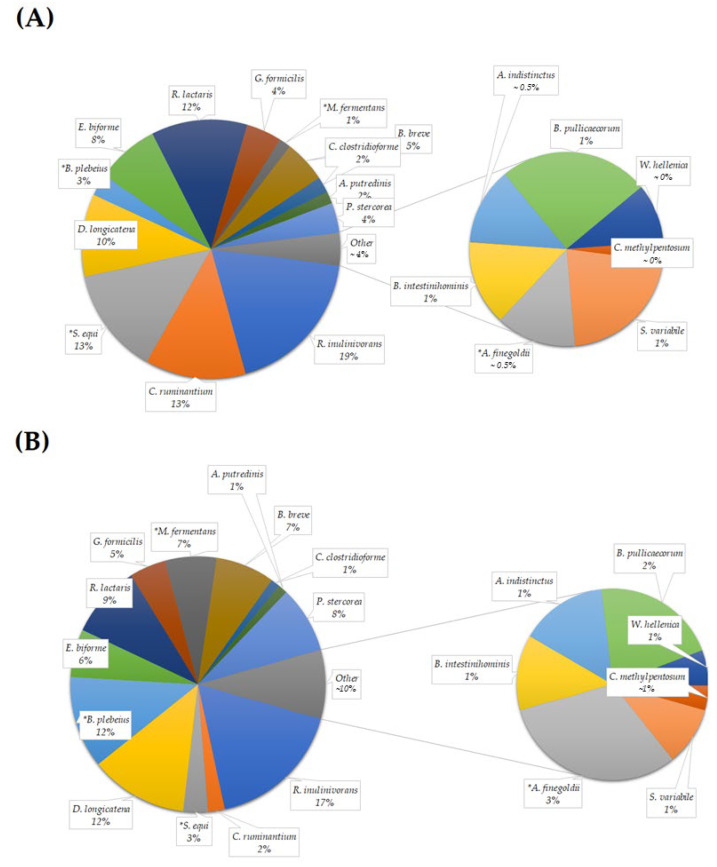
The predominant species in the baseline and treatment samples (≥70% in baseline or treatment) were calculated and compared. Significant differences were calculated for the detected species between the baseline and treatment samples based on the Wilcoxon signed-rank test, with statistical significance *p* ≤ 0.05. Note: *: significantly differed (*p* ≤ 0.05) compared to the baseline samples. (**A**,**B**) represent the values from baseline and treatment samples, respectively.

The species *Roseburia inulinivorans*, *Clostridium ruminantium*, *Streptococcus equi*, *Dorea longecatena*, *Bacteroides plebeius*, *Eubacterium biforme*, *Ruminococcus lactaris*, *Gemmiger formicilis*, *Macellibacteroides fermentans*, *Bifidobacterium breve*, *Clostridium clostridioforme*, *Alistipes putredinis*, *Prevotella stercorea*, *Subdoligranulum variabile*, *Alistipes finegoldii*, *Barnesiella intestinihominis*, *Alistipes indistinctus*, *Butyricicoccus pullicaecorum*, *Weissella hellenica*, and *Clostridium methylpentosum* were detected in both the baseline and treatment samples (Figure 8). Their relative frequencies were tabulated (Table 4). 

A significant reduction was observed in the abundance of *S. equi* (Baseline RF = 4.68 ± 1.51%; Treatment RF = 0.97 ± 0.31%; *p* = 0.028) in the probiotic-treated samples compared to the baseline value. Significant levels of increase were observed in the abundances of *B. plebeius* (Baseline RF = 0.96 ± 0.54%; Treatment RF = 3.66 ± 1.61%; *p* = 0.028), *M. fermentans* (Baseline RF = 0.52 ± 0.18%; Treatment RF = 2.04 ± 0.70%; *p* = 0.037) and *A. finegoldii* (Baseline RF = 0.19 ± 0.06%; Treatment RF = 0.86 ± 0.33%; *p* = 0.047) (Table 4).

### 3.3. Effects of Probiotics on Behavioral Performance

In the visual n-back test, no significant changes were observed in the correct response (*p* = 0.770) and omission error (*p* = 0.826) of target stimuli, or in the correct response (*p* = 0.355) and error of non-target stimuli (*p* = 0.367). Furthermore, there were no statistically significant changes in response time (*p* = 0.244) after 12 weeks of probiotic supplementation (Table 5).

### 3.4. Effects of Probiotics on Brain Activity

In the visual n-back task, mean delta, theta, alpha, beta and gamma waves were calculated at the baseline and after 12 weeks of probiotic supplementation. We did not observe significant changes (delta: *p* < 0.879; theta: *p* < 0.575; alpha: *p* < 0.285; beta: *p* < 0.508; gamma: *p* < 0.959) in any of the waves after probiotic supplementation. 

**Figure 9:**
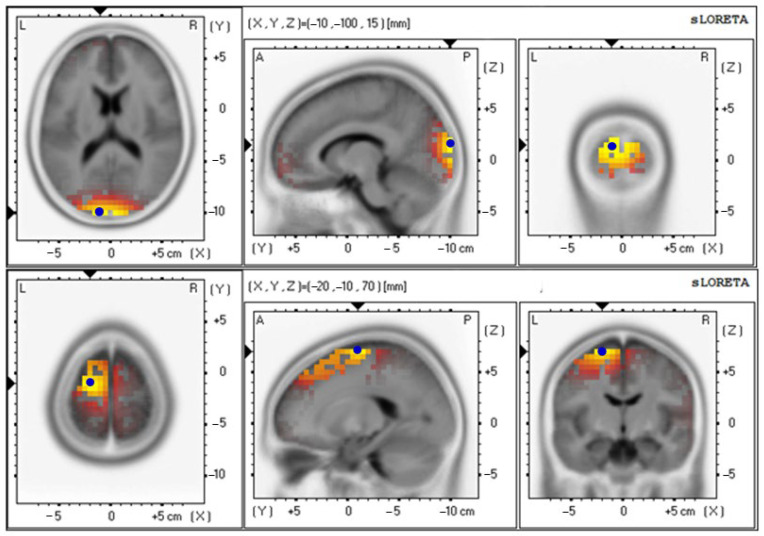
Graphical representation of the standardized Low-resolution Electromagnetic Tomography (sLORETA) *t*-statistic comparing the mean GFP of delta-wave-related stable scalp-potential topography at the cuneus of the left occipital lobe for the baseline (Brodmann area (BA) 18; X = −10, Y = −100, Z = 15; MNI cords; *t* = 4.85) (**top**) and after intervention at the Superior Frontal Gyrus (SFG) of the left frontal lobe (Brodmann area (BA) 6; X = −20, Y = −10, Z = 70; MNI cords; *t* = 4.71) (**bottom**). The yellow areas indicate the local maxima of increased electrical activity in axial, sagittal, and coronal slices through the reference brain. Blue dots mark the center of significantly increased electric activity.

**Figure 10:**
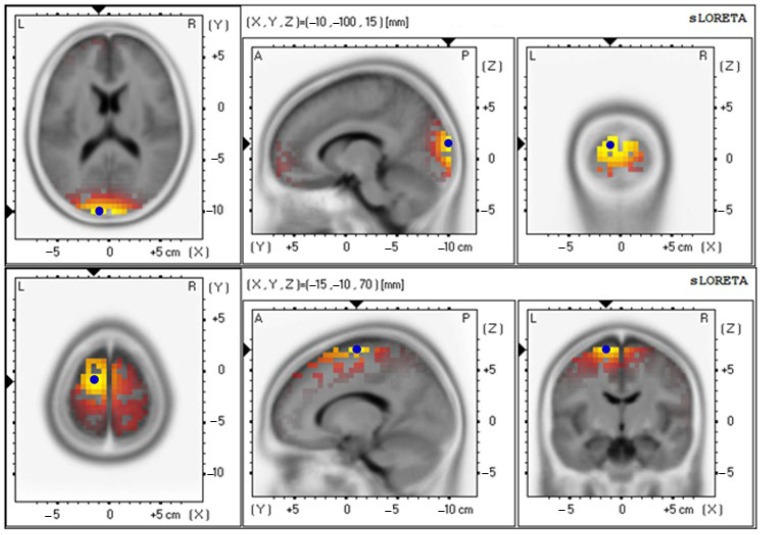
Graphical representation of the standardized Low-resolution Electromagnetic Tomography (sLORETA) *t*-statistic comparing the mean GFP of theta-wave-related stable scalp-potential topography at the cuneus of the left occipital lobe for the baseline (Brodmann area (BA) 18; X = −10, Y = −100, Z = 15; MNI cords; *t* = 3.19) (**top**) and after intervention at the Superior Frontal Gyrus (SFG) of the left frontal lobe (Brodmann area (BA) 6; X = −15, Y = −10, Z = 70; MNI cords; *t* = 3.91) (**bottom**). The yellow areas indicate the local maxima of increased electrical activity in axial, sagittal, and coronal slices through the reference brain. Blue dots mark the center of significantly increased electric activity.

**Figure 11:**
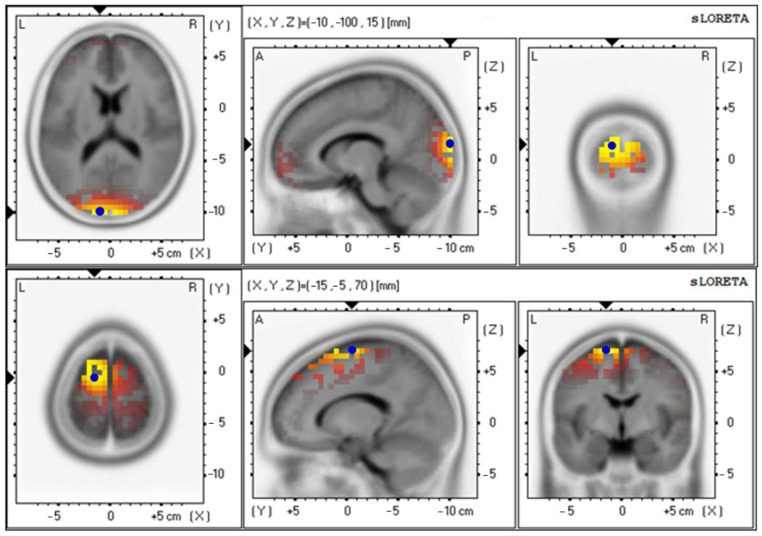
Graphical representation of the standardized Low-resolution Electromagnetic Tomography (sLORETA) *t*-statistic comparing the mean GFP of alpha-wave-related stable scalp-potential topography at the cuneus of the left occipital lobe for the baseline (Brodmann area (BA) 18; X = −10, Y = −100, Z = 15; MNI cords; *t* = 2.66) (**top**) and after intervention at the Superior Frontal Gyrus (SFG) of the left frontal lobe (Brodmann area (BA) 6; X = −15, Y = −5, Z = 70; MNI cords; *t* = 4.05) (**bottom**). The yellow areas indicate the local maxima of increased electrical activity in axial, sagittal, and coronal slices through the reference brain. Blue dots mark the center of significantly increased electric activity.

**Figure 12:**
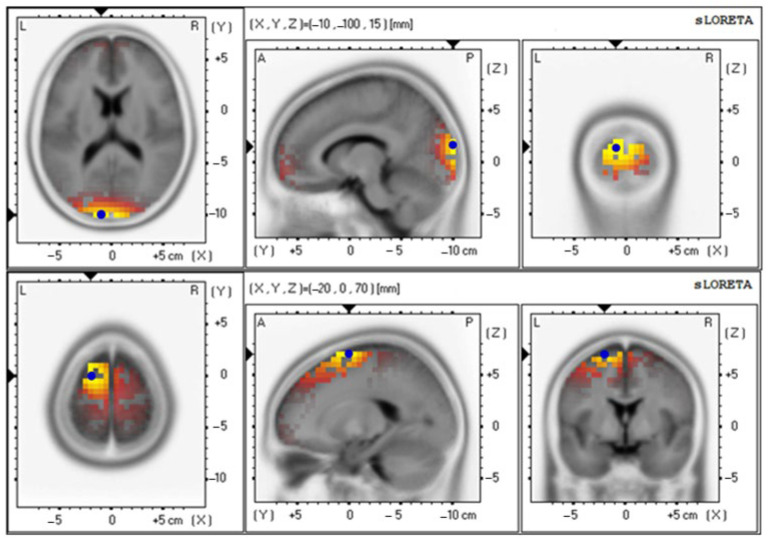
Graphical representation of the standardized Low-resolution Electromagnetic Tomography (sLORETA) *t*-statistic comparing the mean GFP of beta-wave-related stable scalp-potential topography at the cuneus of the left occipital lobe for the baseline (Brodmann area (BA) 18; X = −10, Y = −100, Z = 15; MNI cords; *t* = 4.42) (**top**) and after intervention at the Superior Frontal Gyrus (SFG) of the left frontal lobe (Brodmann area (BA) 6; X = −20, Y = 0, Z = 70; MNI cords; *t* = 3.74) (**bottom**). The yellow areas indicate the local maxima of increased electrical activity in axial, sagittal, and coronal slices through the reference brain. Blue dots mark the center of significantly increased electric activity.

**Figure 13:**
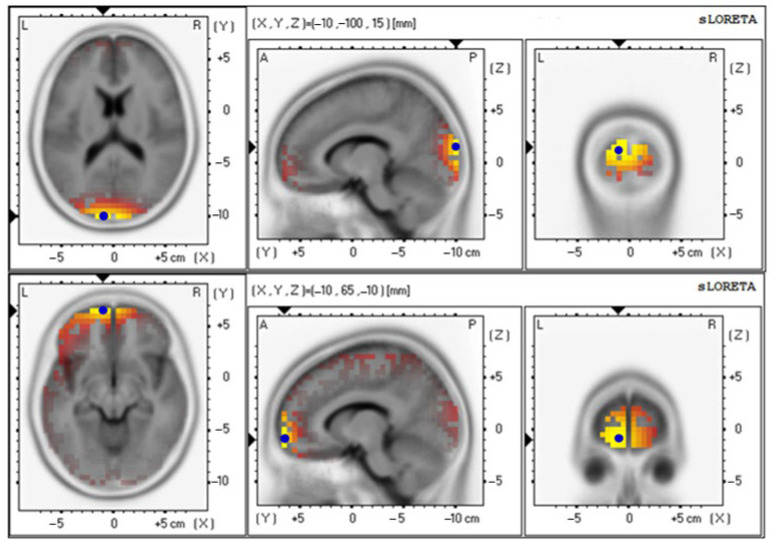
Graphical representation of the standardized Low-resolution Electromagnetic Tomography (sLORETA) *t*-statistic comparing the mean GFP of gamma-wave-related stable scalp-potential topography at the cuneus of the left occipital lobe for the baseline (Brodmann area (BA) 18; X = −10, Y = −100, Z = 15; MNI cords; *t* = 3.69) (**top**) and after intervention at the Superior Frontal Gyrus (SFG) of the left frontal lobe (Brodmann area (BA) 11; X = −10, Y = 65, Z = −10; MNI cords; *t* = 3.70) (**bottom**). The yellow areas indicate the local maxima of increased electrical activity in axial, sagittal, and coronal slices through the reference brain. Blue dots mark the center of significantly increased electric activity.

The baseline and 12th week delta values were 4.85 ± 0.92 and 4.71 ± 0.87, respectively (Figure 9 and Table 6); the baseline and 12th week theta values were 3.19 ± 0.65 and 3.91 ± 0.86, respectively (Figure 10 and Table 6); the baseline and 12th week alpha values were 2.66 ± 0.70 and 4.05 ± 0.89, respectively (Figure 11 and Table 6); the baseline and 12th week beta values were 4.42 ± 0.89 and 3.74 ± 0.87, respectively (Figure 12 and Table 6); the baseline and 12th week gamma values were 3.69 ± 0.70 and 3.70 ± 0.64, respectively (Figure 13 and Table 6).

## 4. Discussion

Obesity is linked with many metabolic disorders, including diabetes, dyslipidemia, cardiovascular diseases, hypertension, and certain types of cancer. Childhood obesity is continuously increasing and becoming a public health burden. Several complementary and adjuvant therapeutic approaches were reported to manage obese conditions, including probiotics [1]. In the present study, the supplementation of probiotics (8 × 10^9^ CFU; *L. paracasei* HII01 and *B. animalis* subsp. *lactis*) for 12 weeks did not significantly improve any of the studied obesity-related parameters (body weight, BMI, body fat, and visceral fat) in obese children, which might indicate that the supplement was insufficient, or the duration was too short to notice the positive changes (Table 2). 

Several studies confirmed the link between gut microbial dysbiosis and conditions like inflammatory diseases and obesity [47,48,49]. SCFAs produced by microbial fermentation from indigestible dietary carbohydrates protect against obesity and metabolic disorders [50]. Acetate, butyrate, and propionate mediate G protein-coupled receptor signaling, enhance insulin sensitivity and satiety, and reduce adipogenesis [50]. In 16S rRNA sequencing studies on obese children, it was revealed that individual variabilities in participants, like their caloric intake and nutrition, might influence the gut microbiota and SCFA concentrations [51]. In the present study, probiotic supplementation significantly increased butyrate and propionate levels (Table 2). The results indicated that the probiotic supplementation altered the gut microbial population.

Fecal indole and cresol, produced by the fermentation of gut microbiota from tryptophan and tyrosine [52], showed reduction after probiotic treatment (Table 2), but no significant changes were found. This suggests that the production of indole and cresol might depend on the concentration and duration of probiotic treatment. Another bioactive metabolite, skatole, was found to be reduced after treatment. The intestinal microbiota play an essential role in synthesizing potential bioactive metabolites, and they may modulate intestinal barrier permeability and immune functions by influencing intracellular signaling [53]. Any higher concentration of indole and/or cresol in the fecal samples signifies higher ammonium levels and lower carbohydrates, representing a pronounced indicator of intestinal proteolytic metabolism. Bacterial taxa *Bacteroides*, *Alistipes*, *Eubacterium*, *Xylanophylum*, and *Barnesiella* are associated with the production of *p*-cresol, while *Bacteroides*, *Ruminococcus torques*, *Blautia*, *Dialister*, and *Butyricicoccus* are associated with the production of indole [52]. However, the levels of indole and cresol might vary depending on individual gut microbial composition and dietary patterns [54]. The changes in the acetic acid, cresol, indole, and skatole levels were insignificant after 12 weeks of probiotic supplementation (Table 2). The changes in the SCFA levels could occur due to the degree of obesity and/or demographic factors such as age, gender, and differences in diet. Thus far, further analysis is required to define the limitations and heterogeneity of this study.

The gut microbiota and its secondary metabolites can affect tryptophan metabolism and gut inflammation, which affect QA levels [55]. Serotonin affects energy balance, appetite, and weight control [56]. Inflammation interferes with serotonin metabolism [55]. 5-HIAA is the product of serotonin involved in the pathogenetic process of obesity, abnormal lipids, and glucose metabolism. Also, 5-HIAA causes low-grade chronic inflammation, resulting in metabolic syndrome [57]. The symbiotic 12-week supplementation (of *L. paracasei*, *B. longum*, *B. breve*, inulin, and fructooligosaccharides) significantly reduced QA levels and QA/5-HIAA ratios in obese Thai adult subjects [58]. The supplementation of a probiotic mixture (*L. paracasei* HII01, *B. longum*, *B. breve*) for 12 weeks significantly reduced QA levels. Still, there were no significant changes in 5-HIAA and QA/5-HIAA ratios in elderly Thai people [58]. The present study results showed that the levels of QA and 5-HIAA and the QA/5-HIAA ratio were significantly altered (Table 2), indicating that the composition of the probiotic supplements and strains might affect the host metabolic activity and inflammatory status.

Oxidative stress occurs because of an imbalance between antioxidants and pro-oxidative factors [59]. Oxidative stress is associated with obesity and progresses with the accumulation of cytotoxic compounds, which promote metabolic changes [60]. We evaluated the TAC and the antioxidant marker MDA in obese children. We found no significant (*p* = 0.114) change in MDA after 12 weeks of probiotic treatment, but a significant level (*p* < 0.001) of improvement was observed in TAC (Table 2).

Gut microbial composition is one of the major factors in metabolic health, anti-inflammatory activities, appetite control, glucose regulation, and lipid metabolism, as well as in the development of obesity; thus, the role of the gut microbiota is of emerging interest in studying obesity [61]. Obese adults and children were found to have altered gut microbiota [62]. In a study of obese individuals, it was found that obese individuals had more Firmicutes and fewer Bacteroidetes than did lean individuals. Obese volunteers who ate a low-fat or low-carbohydrate diet for a year showed fewer Firmicutes than Bacteroidetes. The results showed that nutrient or caloric content alteration can produce changes in gut microbial composition [63]. Children with normal weight have a higher load of *Bifidobacterium* and lower *Staphylococcus aureus* than do obese children [64]. A study between overweight and lean children showed differences in gut microbial composition. A quantitative PCR-based study identified that *Enterobacteriaceae* members were significantly higher in overweight subjects than in lean subjects [65]. *Bacteroides fragilis* and *Lactobacilli* were more prevalent, and *B. vulgatus* were less abundant in children with higher BMIs [66].

In our study, a higher level of butyrate, the by-product of lactate-utilizing bacteria, was found, which signifies the extensive utilization of substrate by gut microbiota in obese children [67]. The butyrate and propionate increase in the treatment phase might be due to butyrate and propionate-producing bacteria. The phyla Firmicutes and Bacteroidetes were more abundant at the baseline. In the case of the treatment phase, the abundance of Firmicutes was reduced, while that of Bacteroidetes was increased after 12 weeks of probiotic treatment (Table 4; Figure 6), indicating that the probiotic intervention might cause changes in the host microbial ecosystem that favor Bacteroidetes. 

The microbial fermentation products acetate, butyrate, and propionate are involved in de novo synthesis and produce additional energy for the body [68]. SCFAs help attenuate inflammatory responses by controlling the production of pro-inflammatory mediators and increasing the production of anti-inflammatory mediators. Butyrate and propionate reduce inflammation by inhibiting IL-6, IL-10, and reactive oxygen species (ROSs) [69]. Several studies focused on the anti-obesity activities of SCFAs in their regulation of the appetite, energy balance, food intake, and various other pathways, including suppressing the lipid synthesis and browning of white adipose tissue [70]. Dietary nutrients, especially dietary fibers, act as substrates for the intestinal microbial community and several microbial species, including the genera *Ruminococcus*, *Bacteroides*, *Roseburia*, *Prevotella*, *Eubacterium*, *Faecalibacterium*, *Bifidobacterium*, *Lactobacillus*, and *Clostridium cluster XIVa*, have been involved in fermenting dietary fibers into different SCFAs [71]. The relative proportion of SCFAs depends on the substrate, microbial composition, and gut transit time [72]. Bacterial species convert lactate and succinate into propionates [73]. Producing propionates [74] and acetates is associated with the abundance of Bacteroides species, while butyrate is associated with that of Firmicutes [6].

Among the members of phylum Firmicutes, abundances in genera such as *Roseburia*, *Faecalibacterium*, *Eubacterium*, *Ruminococcus*, *Gemmiger*, *Subdoligranulum*, and *Coprococcus* were reduced, but significant reductions were observed in *Clostridium* and *Streptococcus*. The abundances of some of the Firmicutes members (i.e., *Dorea*, *Succinispira*, *Butyricicoccus*, and *Weisella*) increased after probiotic intervention, but insignificantly (Table 4). A comparative study between obese and non-obese individuals in the Japanese population revealed that members of Firmicutes, including *Blautia*, *Coprococcus*, *Eubacterium*, *Lactobacillus*, *Ruminococcus*, and *Staphylococcus*, were abundantly present in the microbiota of obese individuals. In contrast, the gut microbiomes of non-obese participants were dominated by *Bacteroides faecichinchillae*, *B. thetaiotaomicron*, *Blautia wexlerae*, *Bulleidia*, *Clostridium boltae*, *Veilonella*, *Oribacterium,* and *Methanobrevibacter smithii* [75].

The abundances of *Bacteroides*, *Prevotella*, *Macellibacteroides*, *Alistipes*, *Barnesiella*, *Butyricimonas*, *Parabacteroides,* and *Paraprevotella* (which belong to the phylum Bacteroidetes) were increased after probiotic treatment. Still, significant changes were observed in *Bacteroides* and *Parabacteroides* (Table 4). Species-level analysis revealed a significant reduction in *S. equi* and increased abundances in *B. plebeius*, *M. fermentans*, and *A. finegoldii* (Table 4).

The increased levels of butyrate and propionate and reduced level of acetate (Table 4) might reflect the microbial composition after the probiotic supplementation. Higher abundances of Bacteroidetes (the propionate producer) and Firmicutes (the butyrate producer) [5] result in increased butyrate and propionate levels. Likewise, the reduced abundance of Actinobacteria (the acetate producer) is reflected in the propionate level. These results explain the relationship between gut microbial differences, in particular, the Firmicutes/Bacteroidetes ratio and the role of these phyla in SCFA production. Some studies report that obese adults have higher SCFA concentrations in their stool than do lean individuals [68,76]. In the present study, the overall concentration of SCFAs was increased after probiotic intervention, which was disputable in the previous reports. The gut microbiome influences neuroinflammation through different pathways. Gut microbiomes regulate several neurodegenerative diseases through neuroinflammation. Microbiome analysis in patients with neurodegenerative disease revealed changes in the fecal microbiome, enhanced pro-inflammatory microbes, and depletion of anti-inflammatory species compared to controls [77]. The intestinal microbiome modulates neuroinflammation by communicating between the brain and the gut through the interaction of microbial metabolites in the intestine, as well as in the peripheral and central nervous systems, and by modulating immune functions. Gut dysbiosis produces inflammation in the brain and intestine, which presents a major risk factor for some CNS diseases and metabolic syndromes [78].

Numerous studies have shown that obese individuals frequently suffer cognitive impairment in specific domains, such as executive function and short-term memory [79,80,81]. Moreover, obese children with partial WM deficits exhibited poor academic performance [82]. Deficits in WM are linked to mental, neurological, and other disorders [83]. The n-back task is widely used in WM assessment tools, affecting various populations, including healthy [84] and aging subjects [85], as well as those with mood conditions [86,87], mild cognitive impairment, and Alzheimer’s disease [88]. 

In the present study, the impact of probiotic consumption on working memory in obese children was studied using the behavioral visual n-back task; the results showed no significant change (Table 5). This may be due to the easiness of the test, i.e., it might be too easy for the participants to memorize and respond correctly. At each level, the presentation order could impact task performance. The task’s difficulty has also been varied by distractor or lure stimuli that appear in the sequence. These stimuli increase interference and demand a stronger mechanism of inhibitory control over irrelevant information and WM processing [89,90]. Hence, the lower difficulty tests are less interference-sensitive than those at higher levels. The percentage of correct targets and correct non-targets, or omission and non-target errors, among the participants did not change significantly during the test (Table 5). It was shown that the participants have an executive process through which to maintain the information at a low-WM load consistently. The relatively simple task does not necessitate continuously updating sequential stimuli [40]. Our findings also aligned with a previous EEG study showing that practice and difficulty in WM tasks promote frontal theta activity [91]. However, there were no detailed reports on the visual n-back assessment in obese Thai children supplemented with probiotics.

Malaguarnera et al. [92] reported that the supplementation of *Bifidobacterium longum* with fructooligosaccharides (FOSs) could improve short-term memory, attention, language, computing ability, and cognitive activities in minimal hepatic encephalopathy patients. Schneider et al. [93] claimed that high-dose, multi-probiotic supplementation improved episodic memory and impacted neuronal mechanisms in major depressive disorder (MDD) symptoms in MDD patients. However, some studies reported that probiotic supplementation did not improve cognitive domains [94]. Probiotic treatment in fibromyalgia patients demonstrated a significant decrease in attention Go/No-Go errors but had no impact on WM performances. It was indicated that the short duration of probiotic treatment was insufficient for impacting WM [95]. The results of the present study also supported the abovementioned statement, particularly in the insignificant changes in brain waves (Table 6) and the visual n-back test.

WM function has traditionally been investigated in terms of two dimensions: within-individual effects of WM load and between-individual differences in task performance. Yang et al. established the n-back task system for objectively evaluating WM ability based on θ power, γ power, and the degree of θ–γ synchronization. The obtained results indicate that accuracy rates might be inappropriate for assessing WM. θ power relates to WM recognition, while γ power varies with the memory load. Moreover, a higher load level would be more appropriate for assessing WM ability. The number of θ–γ3 couplings was highest between the frontal area and Pz and O2 [96].

In human brain imaging, Lamichhane et al. investigated healthy young adults (*n* = 57). The analysis focused on a distinct region of the left lateral prefrontal cortex (LPFC), identified in prior work to show a unique relationship with task performance and WM function. Target accuracy was also independently predicted by the global resting-state connectivity of this LPFC region [97]. Although behavioral transfer effects were not obtained, training was associated with decreased activation in the anterior dorsolateral prefrontal cortex (DLPFC; BA 9/46) while performing the PASAT; this remained stable five weeks later. The changes in the anterior DLFPC largely overlapped with the n-back task fMRI activations. WM training improves efficiency in the brain areas involved in the trained task that may affect untrained tasks, specifically in brain areas responsible for cognitive processes [98].

In addition, several EEG studies have sought to identify the electrophysiological signatures of the n-back task performance. Mahsa et al. revealed significant differences in behavioral and electrophysiological signatures in response to the n-back task [99].

Collectively, studied probiotic supplementation did not significantly improve obesity indexes, but altered SCFA levels, oxidative stress, and neuroinflammatory markers. The results demonstrated the difference in the abundances of bacterial populations and its impact on the bacterial metabolites (SCFAs) in obese children after 12 weeks of specified probiotic supplementation. Further extensive studies (of longer duration and with more study subjects) are needed on the influences of specific probiotic supplementation on obesity indexes, microbiomes, and cognitive improvements.

This study has some limitations, including a very limited sample size (due to the limited number of enrolled subjects), no variation in ethnicity, no placebo group, the short duration of the study, and no follow-up study. Although the study subjects did not report any adverse effects, it is necessary to study the safety of the probiotics in an extended way [100,101]. The shift in the gut microbiota may be transient and temporary [102], as several treatment trials for probiotics have failed to find significant alterations in gut microbiome; individuals may require a longer duration of treatment to have therapeutic effects.

## 5. Conclusions

The supplementation of *L. paracasei* HII01 and *Bifidobacterium animalis* subsp. *lactis* improved gut microbial metabolism, TAC, and neuroinflammation in obese children. The gut microbial composition was changed due to the probiotic supplementation, but not significantly. There were no significant changes in brain waves or in the results of the visual n-back test. A proportional increase in ERP amplitude was observed, suggesting that there are direct subtle changes in brain activity, rather than changes in cognitive test performance. The results of this preliminary study indicated that the intervention’s concentration and/or duration were insufficient for a strong outcome. Further detailed studies using a greater number of subjects, with increased duration and concentration of intervention, might help to identify the role of specified probiotics in physical and psychological improvement in children. 

## Figures and Tables

**Table 1:** General characteristics of the study subjects.

Parameters	Baseline (*n* = 10)
Age (years)	9 *
Gender	
Male, *n* (%)	6 (60.00) **
Female, *n* (%)	4 (40.00) **
Height, cm	139.65 ± 3.26
Body weight, kg	56.02 ± 5.15
Body mass index, kg/m^2^	28.30 ± 1.82
Body fat, %	33.57 ± 3.19
Visceral fat, %	9.60 ± 1.66
Arm circumference, cm	30.42 ± 1.64
Waist circumference, cm	89.28 ± 3.17
Hip circumference, cm	93.30 ± 3.62
Waist/Hip ratio	0.96 ± 0.02

The demographic data are presented as the mean ± standard error of the mean (SEM), or * as median or ** number and percentage.

**Table 2:** Changes in the measured parameters after probiotic supplementation.

Parameters	Probiotic (*n* = 10)		*p*-Value
	Baseline	12 Weeks	
Obesity index			
Body weight, kg	56.02 ± 5.15	56.48 ± 5.31	0.116 ^a^
Body mass index, kg/m^2^	28.30 ± 1.82	28.45 ± 1.87	0.256 ^a^
Body fat, %	33.57 ± 3.19	34.52 ± 3.28	0.115 ^a^
Visceral fat, %	9.60 ± 1.66	9.90 ± 1.73	0.180 ^b^
Gut microbial metabolites			
Butyric acid (µmol/g)	50.24 ± 6.06	87.29 ± 14.36	0.047 ^b^*
Propionic acid (µmol/g)	192.72 ± 42.73	319.79 ± 51.77	0.047 ^b^*
Acetic acid (µmol/g)	32.30 ± 5.17	25.03 ± 2.55	0.241 ^b^
Lactic acid (µmol/g)	25.14 ± 6.64	40.30 ± 7.64	0.066 ^b^
Cresol (µmol/g)	0.28 ± 0.06	0.20 ± 0.06	0.182 ^b^
Indole (µmol/g)	0.28 ± 0.10	0.19 ± 0.04	0.879 ^b^
Skatole (µmol/g)	0.07 ± 0.03	0.04 ± 0.03	0.465 ^b^
Oxidative stress			
TAC (mM)	2.04 ± 0.30	3.01 ± 0.19	<0.001 ^a^*
MDA (µM)	2.26 ± 0.43	1.38 ± 0.26	0.114 ^b^
Neuroinflammation			
Quinolinic acid (ng/mL)	26.77 ± 1.06	15.10 ± 0.57	<0.001 ^a^*
5-HIAA (mg/L)	2.91 ± 0.39	14.91 ± 1.26	<0.001 ^a^*
QA/5-HIAA ratio	0.011 ± 0.001	0.001 ± 0.000	<0.001 ^a^*

* *p* < 0.05 was considered statistically significant. Data are presented as mean ± SE. ^a^ *p*-value obtained from a paired *t*-test. ^b^ *p*-value obtained from the Wilcoxon signed-rank test. TAC: total antioxidant capacity; MDA: malondialdehyde; QA: quinolinic acid; 5-HIAA: 5-Hydroxyindoleacetic acid.

**Table 3:** The 16srRNA amplicon sequences were read, and the pre-processing (which included filtering, denoising, and merging) was conducted to profile the microbial community at the baseline and after 12 weeks of treatment samples.

Sample-Id	Input	Filtered	Denoised	Merged	Non-Chimeric
Baseline					
Baseline-OC01	57,721	49,835	49,496	48,744	47,911
Baseline-OC02	45,004	40,334	40,123	39,633	38,867
Baseline-OC03	44,222	37,735	37,514	36,937	36,239
Baseline-OC04	43,130	37,412	37,151	36,568	33,009
Baseline-OC05	51,124	43,104	42,832	42,179	41,559
Baseline-OC06	44,351	35,481	35,286	34,736	34,558
Baseline-OC07	57,499	41,437	41,311	40,900	40,239
Baseline-OC08	36,116	33,065	32,952	32,602	32,236
Baseline-OC09	50,255	41,731	41,406	40,808	40,736
Baseline-OC10	47,199	43,279	43,081	42,487	42,154
12 weeks					
Treament-OC01	58,045	42,586	42,265	41,461	41,069
Treatment-OC02	46,048	37,025	36,825	36,097	35,665
Treatment-OC03	51,452	40,552	40,247	39,379	38,933
Treatment-OC04	40,492	33,874	33,541	32,704	32,250
Treatment-OC05	49,330	37,242	36969	36,427	35,999
Treatment-OC06	47,891	41,815	41,668	41,180	40,941
Treatment-OC07	53,532	46,425	46,182	45,344	44,892
Treatment-OC08	48,991	40,714	40,492	39,917	39,335
Treatment-OC09	54,958	46,479	46,287	45,612	43,170
Treatment-OC10	49,859	38,559	38,348	37,689	37,603

**Table 4:** The differences in the detected phyla, genera, and species between the baseline (week 0) and after 12 weeks of probiotic supplementation.

Taxonomy	Baseline (Week 0)	After Treatment (Week 12)	*p*-Value
Phylum			
Actinobacteria	14.12 ± 5.25	8.74 ± 1.80	0.721
Firmicutes	62.69 ± 5.09	52.27 ± 5.33	0.235
Bacteroidetes	21.72 ± 5.68	36.00 ± 4.93	0.059
Proteobacteria	1.47 ± 0.71	2.99 ± 1.15	0.286
Genus			
*Collinsella*	6.15 ± 1.90	4.05 ± 1.00	0.445
*Bifidobacterium*	7.45 ± 4.20	5.13 ± 1.95	0.959
*Roseburia*	5.50 ± 2.20	4.40 ± 1.50	0.879
*Bacteroides*	10.39 ± 3.93	22.11 ± 4.23	0.009 *
*Clostridium*	3.23 ± 1.08	0.67 ± 0.12	0.018 *
*Faecalibacterium*	5.87 ± 1.33	4.80 ± 1.19	0.721
*Streptococcus*	4.48 ± 1.26	0.98 ± 0.25	0.017 *
*Prevotella*	9.35 ± 5.57	10.32 ± 4.83	0.647
*Dorea*	3.26 ± 1.11	3.49 ± 1.61	0.508
*Eubacterium*	2.45 ± 1.03	1.57 ± 0.55	0.608
*Ruminococcus*	2.83 ± 0.48	2.49 ± 0.73	0.082
*Gemmiger*	1.26 ± 0.34	1.23 ± 0.40	0.610
*Macellibacteroides*	0.45 ± 0.16	1.70 ± 0.64	0.059
*Alistipes*	0.93 ± 0.30	2.06 ± 0.55	0.114
*Succinispira*	0.13 ± 0.06	1.32 ± 1.14	0.108
*Subdoligranulum*	0.28 ± 0.08	0.25 ± 0.11	0.508
*Barnesiella*	0.16 ± 0.09	0.31 ± 0.17	0.262
*Butyricicoccus*	0.28 ± 0.06	0.50 ± 0.18	0.575
*Weissella*	0.09 ± 0.06	0.12 ± 0.04	0.356
*Butyricimonas*	0.08 ± 0.03	0.39 ± 0.19	0.218
*Coprococcus*	0.28 ± 0.09	0.27 ± 0.10	1.000
*Parabacteroides*	0.04 ± 0.02	0.29 ± 0.14	0.018 *
*Paraprevotella*	0.17 ± 0.07	0.38 ± 0.12	0.126
*Slackia*	0.13 ± 0.06	0.07 ± 0.02	0.356
Species			
*Roseburia inulinivorans*	6.58 ± 2.44	5.42 ± 1.76	0.879
*Clostridium ruminantium*	4.43 ± 2.38	0.67 ± 0.22	0.114
*Streptococcus equi*	4.68 ± 1.51	0.97 ± 0.31	0.028 *
*Dorea longicatena*	3.66 ± 1.24	3.90 ± 1.61	0.879
*Bacteroides plebeius*	0.96 ± 0.54	3.66 ± 1.61	0.028 *
*Eubacterium biforme*	2.78 ± 1.10	1.90 ± 0.71	0.222
*Ruminococcus lactaris*	4.24 ± 0.91	2.90 ± 0.76	0.114
*Gemmiger formicilis*	1.57 ± 0.40	1.44 ± 0.45	0.307
*Macellibacteroides fermentans*	0.52 ± 0.18	2.04 ± 0.70	0.037 *
*Bifidobacterium breve*	1.87 ± 0.82	2.34 ± 1.33	0.799
*Clostridium clostridioforme*	0.70 ± 0.26	0.37 ± 0.09	0.333
*Alistipes putredinis*	0.53 ± 0.32	0.37 ± 0.18	0.758
*Prevotella stercorea*	1.31 ± 0.94	2.65 ± 1.40	0.182
*Subdoligranulum variabile*	0.31 ± 0.10	0.27 ± 0.11	0.445
*Alistipes finegoldii*	0.19 ± 0.06	0.86 ± 0.33	0.047 *
*Barnesiella intestinihominis*	0.20 ± 0.11	0.35 ± 0.19	0.262
*Alistipes indistinctus*	0.19 ± 0.08	0.40 ± 0.19	0.203
*Butyricicoccus pullicaecorum*	0.36 ± 0.09	0.58 ± 0.18	0.575
*Weissella hellenica*	0.14 ± 0.09	0.16 ± 0.07	0.356
*Clostridium methylpentosum*	0.05 ± 0.01	0.11 ± 0.04	0.151

The Wilcoxon signed-rank test was used to determine statistical significance. * Statistical significance at *p* ≤ 0.05.

**Table 5:** Changes in behavioral performance on the visual n-back test within the group at different times.

Parameters	Probiotic (*n* = 10)		*p*-Value
	Baseline	12 Weeks	
correct non-target (%)	93.20 ± 3.44	96.40 ± 1.76	0.355 ^a^
error non-target (%)	6.80 ± 3.44	3.60 ± 1.76	0.367 ^b^
correct target (%)	93.20 ± 3.63	94.00 ± 2.81	0.770 ^a^
omission error (%)	6.80 ± 3.63	6.00 ± 2.81	0.826 ^b^
response time (ms)	501.55 ± 36.28	537.92 ± 34.38	0.244 ^a^

Data are presented as the mean ± SE. ^a^ *p*-value obtained from a paired *t*-test. ^b^ *p*-value obtained from the Wilcoxon signed-rank test.

**Table 6:** The effect of probiotic supplementation on the grand mean global field power (GFP) of electroencephalographic activities within 12 weeks.

Parameters	Electrical Activities (Mean ± SE)	Brodmann Area (BA) (*x*, *y*, *z*)	Lateralization
Delta wave			
Baseline	4.85 *±* 0.92	18 (−10, −100, 15)	Cuneus–Left Occipital Lobe
12th week	4.71 *±* 0.87	6 (−20, −10, 70)	Superior Frontal Gyrus–Left Frontal Lobe
Theta wave			
Baseline	3.19 *±* 0.65	18 (−10, −100, 15)	Cuneus–Left Occipital Lobe
12th week	3.91 *±* 0.86	6 (−15, −10, 70)	Superior Frontal Gyrus–Left Frontal Lobe
Alpha wave			
Baseline	2.66 *±* 0.70	18 (−10, −100, 15)	Cuneus–Left Occipital Lobe
12th week	4.05 *±* 0.89	6 (−15, −5, 70)	Superior Frontal Gyrus–Left Frontal Lobe
Beta wave			
Baseline	4.42 *±* 0.89	18 (−10, −100, 15)	Cuneus–Left Occipital Lobe
12th week	3.74 *±* 0.87	6 (−20, 0, 70)	Superior Frontal Gyrus–Left Frontal Lobe
Gamma wave			
Baseline	3.69 *±* 0.70	18 (−10, −100, 15)	Cuneus–Left Occipital Lobe
12th week	3.70 *±* 0.64	11 (−10, 65, −10)	Superior Frontal Gyrus–Left Frontal Lobe

## Data Availability

Data is contained within the article.
